# Three-Dimensional-Printed Polymer–Polymer Composite Electrolytes for All-Solid-State Li Metal Batteries

**DOI:** 10.3390/polym17172369

**Published:** 2025-08-30

**Authors:** Hao Wang, Xin Xiong, Huie Hu, Sijie Liu

**Affiliations:** 1 Foundation Department, Naval University of Engineering, Wuhan 430033, China; 1920191001@nue.edu.cn (H.W.); 13971444942@163.com (X.X.); 0907042003@nue.edu.cn (H.H.); 2 Research Institute of Tsinghua University in Shenzhen, Shenzhen 518000, China; 3 Institute of Nuclear and New Energy Technology, Tsinghua University, Beijing 100084, China

**Keywords:** all-solid-state lithium batteries (ASSLBs), solid polymer electrolytes (SPEs), 3D printing, polyvinylidene fluoride (PVDF), polyacrylic acid (PAA)

## Abstract

High-performance batteries for military and extreme environment applications require alternatives to conventional liquid lithium-ion batteries (LIBs), which suffer from poor low-temperature performance and safety risks. All-solid-state lithium batteries (ASSLBs) offer enhanced safety and superior low-temperature capability. In this work, we designed and fabricated composite solid-state electrolytes using polyvinylidene fluoride (PVDF) and polyacrylic acid (PAA) as polymer matrices, N,N-dimethylformamide (DMF) as the solvent, and lithium bis(trifluoromethane sulfonimide) (LiTFSI) as the lithium salt. Composite solutions with varying PAA mass ratios were prepared. Advanced three-dimensional (3D) printing technology enabled the rapid and precise fabrication of electrolyte membranes. An ionic conductivity of about 2.71 × 10^−4^ S cm^−1^ at 25 °C, high mechanical strength, and good thermal properties can be achieved through component and 3D printing process optimization. Assembled LiCoO_2_||PVDF@PAA||Li ASSLBs delivered an initial discharge capacity of 165.3 mAh/g at 0.1 mA cm^−2^ (room temperature), maintaining 98% capacity retention after 300 cycles. At 0 °C, these cells provided 157.4 mAh/g initial capacity with 85% retention over 100 cycles at 0.1 mA cm^−2^. This work identifies the optimal PAA ratio for enhanced electrochemical performance and demonstrates the viability of 3D printing for advanced ASSLB manufacturing.

## 1. Introduction

The continuous advancement of military technology imposes increasingly strict demands on battery performance for defense applications, particularly concerning operational capabilities in extreme environments. However, traditional liquid lithium-ion batteries (LIBs), which contain conventional liquid electrolyte systems, exhibit significant performance degradation under low-temperature conditions, such as reduced energy density, decreased charge–discharge efficiency, and shortened cycle life [1,2,3,4]. These limitations become particularly dangerous for military assets such as fighter aircraft, unmanned aerial vehicles (UAVs), and ground combat vehicles operating in frigid climates, severely constraining power supply for propulsion, combat command systems, and battlefield maneuverability. Under low temperatures, the viscosity of the liquid electrolyte increases, leading to reduced mobility of Li^+^. Additionally, low temperatures increase the internal resistance of the battery, converting more energy into waste heat [5]. Furthermore, safety hazards associated with liquid batteries warrant serious attention [6]. The risk of thermal runaway and potential fire incidents in military operational contexts could lead to catastrophic outcomes [7]. Consequently, the development of high-performance battery technologies capable of stable operation under extreme conditions is critically important [8,9,10,11].

As an emerging technological approach, high-performance all-solid-state lithium batteries (ASSLBs) demonstrate notable advantages in low-temperature operation and safety [12,13]. The development of ASSLBs represents a promising strategy to enhance safety and achieve high energy density. This approach replaces flammable liquid electrolytes with solid-state electrolytes (SSEs), enabling the utilization of Li metal anodes (specific capacity ≈ 3860 mAh g^−1^) [14,15,16,17]. Compared to traditional liquid LIBs, ASSLBs not only maintain higher energy density and stable discharge characteristics in cold environments but also effectively mitigate safety risks [17,18,19]. Unlike traditional liquid electrolytes, SSEs do not freeze due to low temperatures, thus ensuring the normal operation of the battery in cold environments [20,21]. Moreover, the ionic transport performance of SSEs is stable and is not affected by fluctuations in environmental temperature. Even at low temperatures, they can maintain efficient ionic conduction [22,23]. At the same time, the chemical structure of SSEs is stable, avoiding the structural deformation problems that may occur in liquid electrolytes at low temperatures, further ensuring the stability of the battery’s performance [24,25]. Importantly, the inherent structural properties of ASSLBs confer superior resistance to shock and vibration. These enhanced properties make them significantly better suited to withstand the harsh demands of battlefield environments [26,27].

SSEs can be categorized into solid inorganic electrolytes (SIEs) and solid polymer electrolytes (SPEs) [28,29]. SIEs are fast ion conductors such as oxide [30,31,32], sulfide [33,34,35], and halide [36,37]. They can offer significant advantages including high ionic conductivity, excellent mechanical properties, and inherent safety [38,39,40,41]. However, their practical application faces challenges primarily due to poor interfacial contact with electrodes and difficulties in processing and fabrication. SPEs, in contrast, have attracted much research interest in the SSE field [42]. This is owing to their superior flexibility, favorable mechanical properties, improved electrode/electrolyte interfacial compatibility, and relatively easy processability [43]. Among polymer matrices, poly(vinylidene fluoride) (PVDF) stands out [44,45]. Its high dielectric constant effectively promotes lithium salt dissociation. PVDF also demonstrates a valuable combination of good mechanical flexibility, adequate mechanical strength, robust thermal stability, and a wide, stable electrochemical window. These properties jointly make PVDF a highly promising material for advanced SPE research. For example, Zhang et al. prepared PVDF-based SPEs and discovered that an in situ-formed nanoscale mosaic interface layer (≈20 nm thick, rich in LiF and sulfur compounds) between the PVDF and lithium bis(trifluoromethane sulfonimide) (LiTFSI) electrolyte and Li anode effectively suppresses lithium dendrite growth [46]. This unique interface enables exceptional cycling stability (>2000 h in Li||Li symmetric cells) and induces an “open-circuiting” feature at high current densities instead of dangerous short-circuiting, significantly enhancing the safety and performance of LiCoO_2_||Li batteries. However, the ionic conductivity of single-component SPEs is insufficient for practical applications. Consequently, researchers have fabricated polymer–polymer composite SPEs by combining two polymers to enhance performance [47]. Huang et al. studied a PVDF-derived polymer–polymer composite SPE formed by coupling a high-dielectric-constant terpolymer (P(VDF-TrFE-CTFE)) with an all-trans conformation copolymer (P(VDF-TrFE)) [48]. This PVDF-based composite matrix simultaneously enhanced lithium salt dissociation and constructed efficient Li^+^ transport highways via aligned fluorine atoms, achieving high ionic conductivity (2.37 × 10^−4^ S cm^−1^) and Li^+^ transference number (0.61) at 25 °C, enabling ultralong cycling (>4500 h) in Li symmetric cells. This work demonstrates the effectiveness of organic component coupling for designing high-performance PVDF-based SPEs. Therefore, it is important to construct higher-performance PVDF-based SPEs using a polymer–polymer composite system.

Nowadays, ASSLBs confront significant commercialization barriers due to the high cost and scalability limitations inherent in conventional manufacturing methods [49]. While additive manufacturing technologies are established across the industry, their adaptation for advanced energy storage systems, particularly ASSLBs, remains underdeveloped. Current research heavily prioritizes SSE material innovation but relies on traditional processes. These methods exhibit drawbacks, including inefficient material usage, constraints in structural complexity, and limited capacity for complex design or customized production, ultimately falling short of meeting the energy density and performance requirements of next-generation ASSLBs. Three-dimensional printing (3D printing), as a form of advanced manufacturing, can bring many advantages to the production of SSEs and ASSLBs [50,51]. This technology provides precise control over the battery’s internal structure, thus mitigating internal short-circuit risks. Furthermore, it enables optimization of electrode and electrolyte geometries, shortening ion transport pathways and enhancing energy density. Eliminating complex tooling reduces production setup time and costs, while rapid prototyping accelerates development cycles. It is worthwhile mentioning that achieving higher volumetric energy density requires thinner SPE membranes, yet the traditional solution casting method typically produces SPEs exceeding 100 μm in thickness. Nevertheless, 3D printing offers a viable alternative to produce substantially thinner SPE films. Combined with the numerous advantages of 3D printing, such as design flexibility and cost optimization, it is highly necessary to develop 3D-printed SPE membranes.

In this work, we prepared a 25 μm polymer–polymer composite SPE membrane via 3D printing, using PVDF as the matrix incorporated with a small amount of polyacrylic acid (PAA). The composite SPE with 3 wt% PAA exhibited a room-temperature ionic conductivity of 2.71 × 10^−4^ S/cm, demonstrating significantly enhanced ion transport. Structural characterization confirmed reduced PVDF crystallinity and improved lithium-ion mobility while maintaining thermal stability. When deployed in LiCoO_2_||PVDF@PAA||Li ASSLBs, the optimized composite SPE delivered an initial discharge capacity of 165.3 mAh/g at 0.1 mA cm^−2^ at room temperature, retaining 98% capacity after 300 cycles. At 0 °C, the batteries achieved 157.4 mAh/g initial capacity with 85% retention over 100 cycles. The enhanced performance benefits from uniform lithium salt distribution and optimal ionic conduction at moderate PAA loading, whereas excessive PAA caused conductivity reduction (e.g., 1.99 × 10^−4^ S/cm when PAA content is 20 wt%) due to salt aggregation. This study validates 3D-printed ultrathin composite SPEs for extreme condition ASSLBs.

## 2. Materials and Methods

### 2.1. Materials

#### Preparation of SPEs

PVDF (Aladdin Scientific, Riverside, CA, USA, 99.9%), PAA (Aladdin Scientific, 99.9%), and LiTFSI (Aladdin Scientific, 99.9%) were stored in a glovebox containing argon. PVDF and LiTFSI (weight ratio 3.5:1) were first dissolved in 26 mL of N,N- dimethylformamide (DMF) (Aladdin Scientific, 99.8%) and mechanically stirred for 6 h to obtain a uniform solution. PAA was then added into the homogeneous solution with the PAA weight percentage of 0, 1, 3, 5, and 20 wt% in the total amount of PVDF and PAA. Then, it was mechanically stirred for 12 h to disperse evenly. At last, it stood for another 6 h to obtain a printable slurry. After that, the electrolyte liquid film was obtained by 3D printing via a direct ink writing (DIW) system. Finally, the composite SPE membranes (~25 µm in thickness) were obtained by further drying in a vacuum oven at 60 °C for 24 h to remove traces of the DMF solvent.

### 2.2. Material Characterization

X-ray diffraction (XRD) patterns were collected on an Ultima IV (Cu*Kα) diffractometer operated at 40 kV and 40 mA. Scanning electron micrograph (SEM) images were obtained with an Axia ChemisEM HiVac, Thermo Fisher Scientific (Waltham, MA, USA) tungsten filament scanning electron microscope. The stress and strain curves of the SPE membranes with a size of ~16 mm × 40 mm × 0.1 mm were recorded by a Zwick testing machine at a stretching speed of 10 mm min * 1 at room temperature. The Fourier transform infrared (FTIR) spectra were acquired on a Thermo Fisher Scientific Brno s.n.o. The stress–strain curve of CPE film with dimensions of ~16 mm × 40 mm × 0.1 mm was recorded by a tension machine (HD-B604-S, Dongguan Haida Instrument Company, Dongguan, China) at room temperature with a tensile speed of 10 mm/s.

### 2.3. Electrochemical Measurement

The sandwich cell of SS (stainless steel)/SPE membrane/SS was used for the test of ionic conductivity, which was performed by an impedance analyzer (CORRTEST CS350H) over the frequency range of 1 Hz to 1 MHz. The electrochemical performance of the SPE membranes was evaluated based on CR2025 coin-type cells, which were assembled with a LiCoO_2_ composite cathode, Li tablet anode, and SPE membrane as both electrolyte and separator. The cell was fabricated in an Ar-filled glove box (O_2_ < 5 ppm, H_2_O < 5 ppm). The charge–discharge and cycling properties were tested under galvanostatic conditions between 3 V and 4.2 V using a battery test system (CT3003K, Lanhe Technology Co., Ltd., Shenzhen, China).

## 3. Results and Discussion

**Figure 1:**
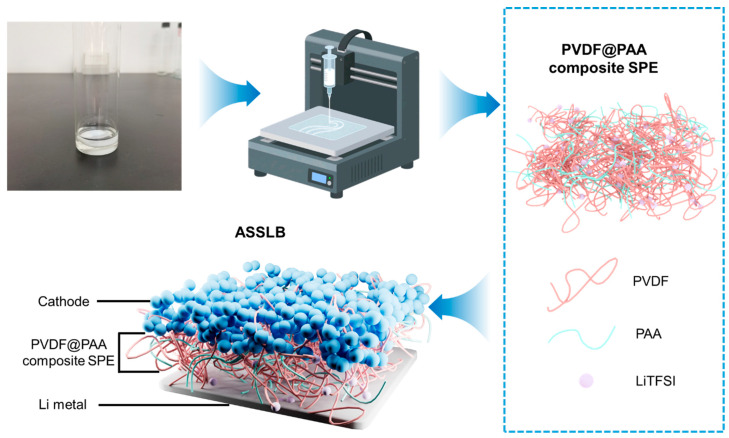
Schematic illustration of the preparation process for the 3D printing of PVDF@PAA composite SPE membranes. The photo in the top-left corner shows the slurry used for 3D printing.

Figure 1 schematically illustrates the preparation step for the 3D printing of PVDF@PAA-based composite SPE membranes. By optimizing the ratios of PAA, PVDF, DMF, and lithium salt, and adjusting the PAA percentage in composite SPEs, a homogeneous slurry was prepared, as shown in the photograph in the top-left corner of Figure 1 (specific experimental procedures can be seen in the Experimental section) [52]. The prepared composite electrolyte slurry was used for 3D printing. Before printing, the program was set using 3D printing software. An air pump adjusted the printer pressure so the ink flowed steadily from the syringe. Different composite SPE films with different PAA contents of 0, 1, 3, 5, and 20 wt% were successfully fabricated. In the PVDF@PAA composite SPE, PVDF and PAA are mixed together to form a network, facilitating the transport of lithium ions from LiTFSI.

**Figure 2:**
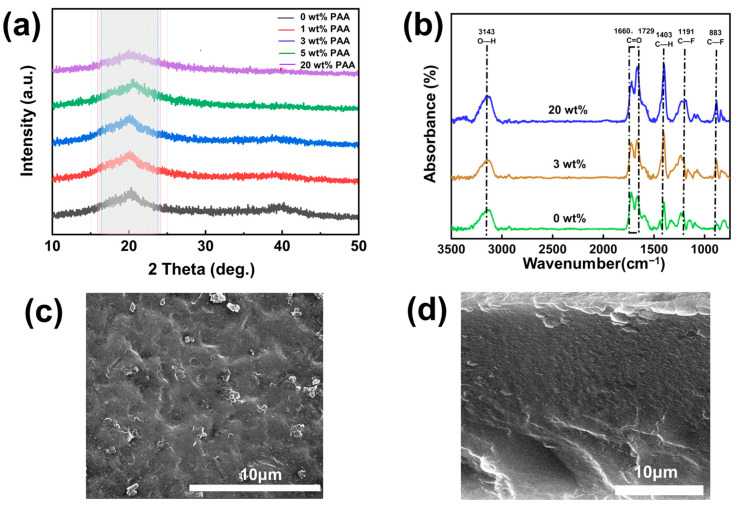
(**a**) XRD patterns of PVDF SPE and PVDF@PAA composite SPEs with different PAA contents (1 wt%, 3 wt%, 5 wt%, and 20 wt%). (**b**) FTIR spectra of PVDF SPE and PVDF@PAA composite SPEs with different PAA contents (3 wt% and 20 wt%). (**c**) Top-view and (**d**) cross-sectional SEM image for the PVDF@PAA composite SPEs with 3 wt% PAA.

According to the X-ray diffraction (XRD) patterns in Figure 2a, as the PAA loading increases, the full width at half maximum (FWHM) of the diffraction peaks for the composite SPE exhibits an increasing trend. For example, the composite PVDF@PAA SPE with 3 wt% PAA has an FWHM of 8.36, while the SPE without PAA has an FWHM of 6.49. A smaller FWHM indicates a larger crystallite size and better crystalline integrity. This indicates that dispersing PAA in PVDF reduces PVDF crystallinity, enhancing lithium salt dissolution and lithium-ion migration. Figure 2b shows the Fourier transform infrared spectroscopy (FTIR) spectra of PVDF SPE and PVDF@PAA composite SPEs with different PAA contents (3 wt% and 20 wt%). The peak at 3143 cm^−1^ corresponds to the O-H stretching vibration, primarily originating from the carboxyl groups in PAA. The peak at 1729 cm^−1^ represents the C=O stretching vibration of the carboxyl group [53]. Due to the abundance of methylene groups in the structure, the peaks observed at 1403 cm^−1^ can both be assigned to C-H bending vibrations. The peaks at 1191 cm^−1^ and 883 cm^−1^ correspond to the C-F stretching vibration and C-F bending vibration of PVDF, respectively. Increasing the PAA content does not significantly change the types of peaks, indicating no significant change in the composite SPEs. However, noticeable changes in peak intensity occur. At a 20 wt% PAA loading, the intensity of peaks associated with PAA (C=O, O-H) increases. Furthermore, the increased PAA content alters the composition of the composite. This change in composition is reflected in a decreased relative intensity of the characteristic C-F peak of PVDF at 1191 cm^−1^. These results demonstrate the successful formation of the PVDF/PAA composite system in SPEs. The scanning electron microscopy (SEM) image of the composite SPE further proves that point. Figure 2c,d show a top-view and cross-sectional SEM image for the PVDF@PAA composite SPEs with 3 wt% PAA. It can be seen that both the surface and cross-section views reveal a dense structure in the composite SPE membrane. No obvious phase separation is observed, indicating effective integration between PVDF and PAA. This dense morphology highlights the advantages of the 3D printing fabrication process. Notably, the membrane maintains excellent structural uniformity despite its ultra-thin thickness of 25 μm. This demonstrates that 3D printing can produce ultra-thin, dense SPE membranes, which are highly beneficial for ion transport and performance enhancement in corresponding ASSLBs.

**Figure 3:**
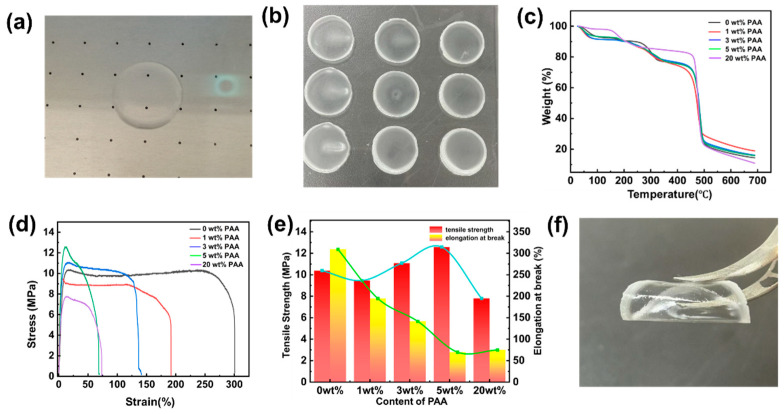
The photo of the 3D-printed PVDF@PAA composite SPE membrane (**a**) before and (**b**) after drying. (**c**) TGA curves of PVDF SPE and PVDF@PAA composite SPEs with different PAA contents (1 wt%, 3 wt%, 5 wt%, and 20 wt%). (**d**) Stress−strain curves of PVDF SPE and PVDF@PAA composite SPEs with different PAA contents (1 wt%, 3 wt%, 5 wt%, and 20 wt%). (**e**) Tensile strength and elongation at break of PVDF SPE and PVDF@PAA composite SPEs with different PAA contents (1 wt%, 3 wt%, 5 wt%, and 20 wt%). (**f**) Photo of the flexibility of the PVDF@PAA composite SPE.

The 3D-printed composite SPE is transparent (Figure 3a,b). The photo of 3D-printed PVDF@PAA composite SPEs with different PAA contents (0 wt%, 1 wt%, and 3 wt%) can be seen in Figures S1–S3. Good thermal stability is essential for practical applications of SPEs in ASSLBs. Figure 3c shows the thermogravimetric analysis (TGA) curves of PVDF SPE and PVDF@PAA composite SPEs with different PAA contents (1 wt%, 3 wt%, 5 wt%, and 20 wt%). No marked difference in thermal stability is observed between PVDF SPE and PVDF@PAA composite SPEs; all electrolytes display high thermal stability. Weight loss observed below 150 °C is attributed to absorbed moisture in the electrolytes. The mechanical robustness of SPEs is essential for ASSLB applications. Figure 3d,e details the Young’s modulus, tensile strength, and elongation at break measured for these PVDF@PAA composite SPEs. The PAA doping concentration exerts a significant influence on the mechanical characteristics of PVDF-based composite electrolyte membranes. The pure PVDF SPE (0 wt% PAA) demonstrates a peak stress of 10.5 MPa coupled with an exceptional elongation at break of 310%, highlighting its notable strength–toughness synergy. A marked shift in mechanical behavior occurs at 1 wt% PAA, peak stress decreases to 9.5 MPa, while elongation at break plummets to 200%. This indicates that even minimal PAA incorporation compromises the membrane’s mechanical integrity. Notably, a nonlinear mechanical response emerges at 3 wt% PAA. Although tensile strength recovers slightly to 11.5 MPa, elongation continues declining to 140%. This suggests that the incorporation of PAA chains may contribute to the strength recovery through composite formation, but insufficiently counteracts the detrimental effect of interfacial defects on toughness. At 5 wt% PAA, peak stress further increases to 12.5 MPa, but elongation drastically drops to 70%. This decoupling of strength and toughness confirms a brittle transition induced by excessive PAA. Ultimately, the 20 wt% PAA sample exhibits comprehensive mechanical deterioration. The low peak stress (7.5 MPa) and minimal elongation (70%) collectively indicate that high PAA loading disrupts the continuous PVDF matrix, promoting severe stress concentration and crack propagation. Thus, pure PVDF membranes exhibit high strength and toughness owing to their substantial crystallinity and strong intermolecular forces. The introduction of PAA disrupts the PVDF crystalline structure and weakens intermolecular interactions, leading to reductions in both strength and ductility. It can be seen from the photo in Figure 3f that the PVDF@PAA composite SPE is flexible and can be bent, which proves its excellent flexibility (more photos can be seen in Figure S4).

**Figure 4:**
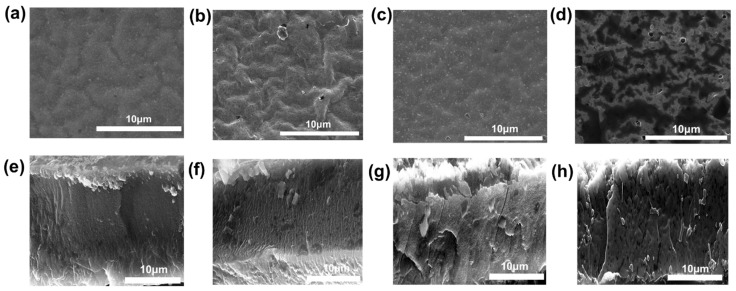
Top-view SEM images for the (**a**) PVDF SPE and (**b**–**d**) PVDF@PAA composite SPEs with different PAA contents (1 wt%, 5 wt%, and 20 wt%). Cross-sectional SEM images of (**e**) the PVDF SPE and (**f**–**h**) PVDF@PAA composite SPEs with different PAA contents (1 wt%, 5 wt%, and 20 wt%).

Figure 4a–d and Figure 3c depict top-view SEM images of PVDF@PAA composite SPE membranes with varying PAA compositions. Figure S5 shows corresponding low-resolution SEM images. All membranes demonstrate highly uniform morphologies across the series. Nevertheless, the morphology of the composite SPEs reveals a small transformation in the membrane surface structure with increasing PAA content. The undoped PVDF membrane (0 wt% PAA) exhibits uniformly distributed particles with consistently small pore sizes. As PAA content rises, subtle textural variations emerge, accompanied by a slight reduction in particle distribution uniformity and minor pore size variations. A distinct transition occurs at intermediate PAA concentrations (3 wt% and 5 wt%). Particle distribution becomes increasingly heterogeneous, accompanied by significant pore size disparities and localized formation of larger pores. This trend culminates in the 20 wt% PAA membrane, which displays markedly irregular surface morphology. Particle distribution is highly non-uniform, pore sizes exhibit substantial variation, and large pores are evident. Figure 4e–h show cross-sectional SEM images of the PVDF SPE and PVDF@PAA composite SPEs with different PAA contents (1 wt%, 5 wt%, and 20 wt%), respectively. Cross-sectional SEM images confirm consistent membrane thickness (~25 μm) of all SPEs. As can be seen in Figure S6, the photo of the thickness test for 3D-printed PVDF@PAN membranes with 3 wt% PAA further proves its thickness is about 25 μm. While increased PAA content enhances interactions with PVDF, causing minor film contraction, this dimensional change is negligible for practical purposes. Significantly, all composite electrolyte membranes exhibit exceptionally dense cross-sections. This characteristic further proves the precision and capability of the 3D printing fabrication.

**Figure 5:**
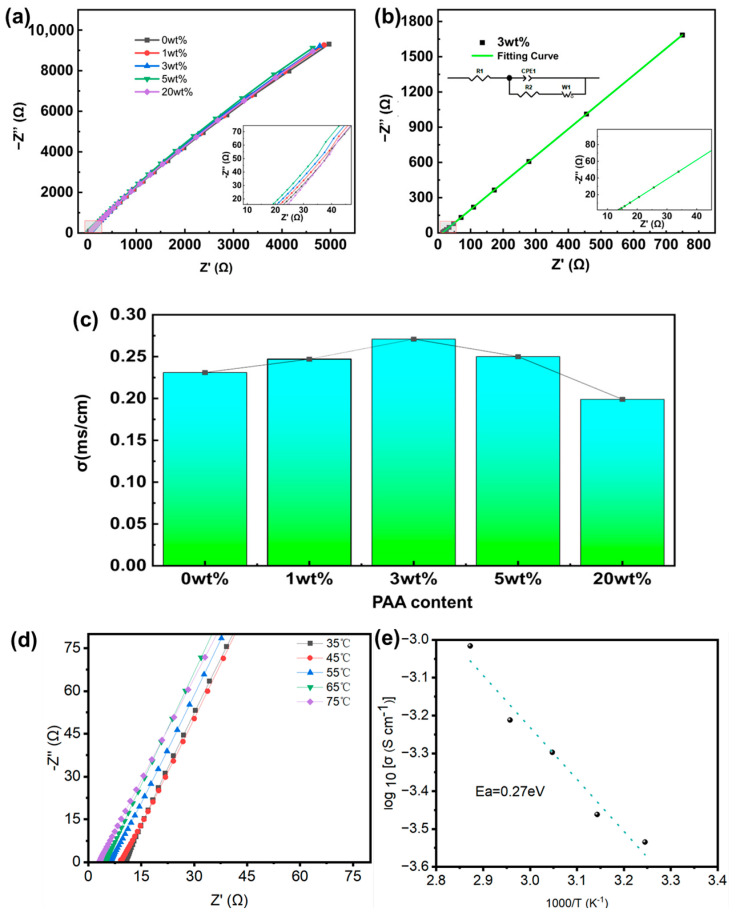
(**a**) Nyquist plots of the PVDF SPE and PVDF@PAA composite SPEs with different PAA contents (1 wt%, 3 wt%, 5 wt%, and 20 wt%). (**b**) Nyquist plot and corresponding fitting curve of PVDF@PAA composite SPE with 3 wt% PAA. (**c**) The relationship between ionic conductivity and the content of PAA for PVDF@PAA composite SPEs. (**d**) Nyquist plots of the PVDF@PAA composite SPE with 3 wt% PAA at 35–70 °C. (**e**) Arrhenius plot of the ionic conductivity of the PVDF@PAA composite SPE with 3 wt% PAA.

The Nyquist plots of PVDF SPE and PVDF@PAA composite SPEs with different PAA contents (1 wt%, 3 wt%, 5 wt%, and 20 wt%) measured at room temperature are presented in Figure 5a. The ionic resistance R_1_ and charge transfer resistance R_2_ of the batteries are determined by fitting the Nyquist plot with an equivalent circuit, which is shown in Figure S7. R_1_ corresponds to the combined resistance of the electrolyte and electrode layers, while *R*_2_ primarily represents the interfacial resistance between the electrodes and electrolyte [54]. The Nyquist plots and corresponding fitting curve of PVDF SPE and PVDF@PAA composite SPEs with different PAA contents (1 wt%, 3 wt%, 5 wt%, and 20 wt%) are shown in Figure 5b and Figure S8–S11. Table 1 shows the fitting results of impedance spectra and the corresponding ionic conductivity for PVDF SPE and PVDF@PAA composite SPEs with different PAA contents (1 wt%, 3 wt%, 5 wt%, and 20 wt%). The results indicate that the impedance behavior of the composite SPEs changes systematically with increasing PAA content. Within the low PAA concentration range, both R_1_ and R_2_ of the SPE decrease progressively with higher PAA loading. This reduction demonstrates that incorporating modest amounts of PAA effectively enhances ionic conductivity within the PVDF matrix, thereby lowering the overall electrolyte impedance. The improvement likely stems from interactions between PAA and PVDF that disrupt crystalline order, expanding amorphous domains, and creating additional pathways for lithium-ion transport. Furthermore, carboxyl groups in PAA interact with lithium ions from the salt, facilitating their dissociation and mobility. When the PAA content is 3 wt%, the composite SPE shows the lowest R_1_ and R_2_ as 12.21 Ω and 17.63 Ω, respectively. The ionic conductivity σ was calculated by the equation σ = L/RS, where R is the bulk resistance obtained by the simulation according to the equivalent circuit and L and S are the thickness and area of the SPE membrane, respectively. The composite SPE with 3 wt% PAA shows the highest ionic conductivity of 2.71 × 10^−4^ S cm^−1^ at room temperature. Conversely, impedance values increase significantly at higher PAA concentrations (5 wt% and 20 wt%). This degradation suggests excessive PAA content adversely impacts ionic conductivity. Potential mechanisms include elevated system viscosity restricting ion migration, or excessively strong PAA–lithium salt interactions promoting the formation of large ionic aggregates that impede lithium-ion conduction. Figure 3c shows the relationship between ionic conductivity and the content of PAA for PVDF@PAA composite SPEs. The ionic conductivity initially increases with PAA loading, rising from 2.31 × 10^−4^ S/cm for pure PVDF (0 wt% PAA) to a maximum of 2.71 × 10^−4^ S/cm at 3 wt% PAA. However, further increasing the PAA content to 5 wt% and 20 wt% results in a decline in conductivity, with values falling to 2.50 × 10^−4^ S/cm and 1.99 × 10^−4^ S/cm, respectively. Figure 5d shows the Nyquist plots of the PVDF@PAA composite SPE with 3 wt% PAA at 25–70 °C. Correspondingly, the Arrhenius plot of the PVDF@PAA composite SPE with 3 wt% PAA is shown in Figure 5e. The activation energy *E_a_* was calculated according to the classical Arrhenius equation σ(T) = A exp(−*E_a_*/RT), where T is the absolute temperature and A is a pre-exponential factor. *E_a_* is calculated to be about 0.27 eV.

**Figure 6:**
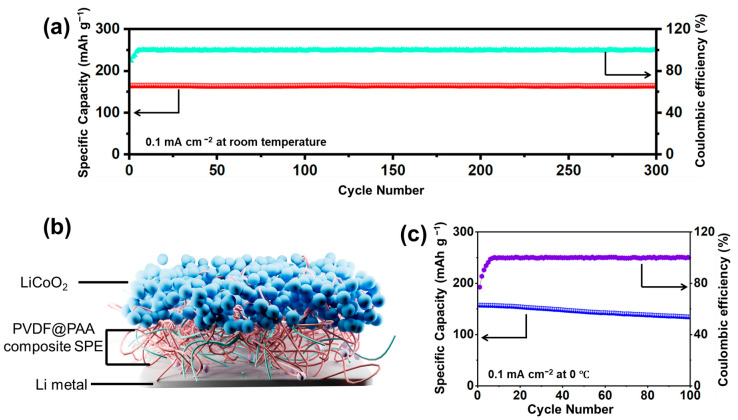
(**a**) Electrochemical performances at room temperature for LiCoO_2_||PVDF@PAA||Li ASSLB with 3 wt% PAA. (**b**) Structure diagram LiCoO_2_||PVDF@PAA||Li ASSLB. (**c**) Electrochemical performances at 0 °C for LiCoO_2_||PVDF@PAA||Li ASSLB with 3 wt% PAA.

To evaluate the practical utility of the SPE membranes, full cells were fabricated using the optimized composite PVDF@PAA membrane (3 wt% PAA) as the electrolyte layer, paired with a LiCoO_2_ cathode and lithium metal anode (Figure 6b). As shown in Figure 6a, the resulting LiCoO_2_||PVDF@PAA||Li ASSLBs delivered an initial discharge capacity of 165.3 mAh g^−1^ at 0.1 mA cm^−2^ under ambient conditions, retaining 98% capacity after 300 cycles. At 0 °C and the same current density, these ASSLBs provided 157.4 mAh g^−1^ initial capacity with 85% retention over 100 cycles (Figure 6c). This not only identifies 3 wt% PAA as the optimal composition for enhanced electrochemical performance but also validates the viability of 3D-printed composite electrolytes for advanced ASSLB applications.

## 4. Conclusions

A series of ultrathin (25 μm) polymer–polymer composite SPEs were synthesized via 3D printing, employing PVDF as the matrix with systematically varied PAA content (0–20 wt%). Among these composite PVDF@PAA SPEs, the 3 wt% PAA composite SPE demonstrated optimal performance, exhibiting a room-temperature ionic conductivity of 2.71 × 10^−4^ S/cm (a 17% enhancement over pure PVDF). Structural analyses confirmed that this composition optimally suppressed PVDF crystallinity while enhancing lithium-ion mobility and maintaining thermal stability. When integrated into LiCoO_2_||PVDF@PAA||Li ASSLBs, it delivered an initial discharge capacity of 165.3 mAh g^−1^ at 0.1 mA cm^−2^ under room temperature, with 98% capacity retention after 300 cycles. Critically, at 0 °C as the low temperature, LiCoO_2_||PVDF@PAA||Li ASSLBs maintained 157.4 mAh g^−1^ initial capacity and 85% retention over 100 cycles at the same 0.1 mA cm^−2^. These performance metrics substantially surpass conventional PVDF-based electrolytes under subzero conditions. The performance enhancement stems from uniform lithium salt distribution and optimized ion-conduction pathways at moderate PAA loading. Conversely, excessive PAA (e.g., 20 wt%) reduced conductivity to 1.99 × 10^−4^ S/cm due to salt aggregation and disrupted ion mobility. This study establishes 3D printing as a viable strategy for manufacturing advanced ultrathin composite SPEs, demonstrating their exceptional applicability in extreme condition ASSLBs.

## Figures and Tables

**Table 1:** The fitting results of impedance spectra and the corresponding ionic conductivity for PVDF SPE and PVDF@PAA composite SPEs with different PAA contents (1 wt%, 3 wt%, 5 wt%, and 20 wt%).

PAA Content	R_1_ (Ω)	R_2_ (Ω)	Ionic Conductivity (S/cm)
0 wt%	13.75	18.62	2.31 × 10^−4^
1 wt%	12.89	18.16	2.47 × 10^−4^
3 wt%	12.21	17.63	2.71 × 10^−4^
5 wt%	12.74	17.99	2.50 × 10^−4^
20 wt%	15.31	20.56	1.99 × 10^−4^

## Data Availability

The data presented in this study are available on request from the corresponding author. The data are not publicly available due to privacy restrictions.

## Supplementary Materials

The following supporting information can be downloaded at https://www.mdpi.com/article/10.3390/polym17172369/s1, Figure S1: The photo of 3D-printed PVDF membranes; Figure S2: The photo of 3D-printed PVDF@PAN membranes with 1wt% PAA; Figure S3: The photo of 3D-printed PVDF@PAN membranes with 3wt% PAA; Figure S4: The photo of bendable 3D-printed PVDF@PAN membranes with 3wt% PAA; Figure S5: (a)-(e) SEM images of PVDF SPE and PVDF@PAN composite SPEs with different PAA contents (1wt%, 3wt%, 5wt% and 20wt%); Figure S6: The photo of thickness test for 3D-printed PVDF@PAN membranes with 3wt% PAA; Figure S7: The equivalent circuit used in impedance spectrum fitting; Figure S8: Nyquist spectra and corresponding fitting curve of PVDF SPE (namely 0wt% PAA); Figure S9: Nyquist spectra and corresponding fitting curve of PVDF@PAN composite SPE with 3wt% PAA; Figure S10: Nyquist spectra and corresponding fitting curve of PVDF@PAN composite SPE with 5wt% PAA; Figure S11: Nyquist spectra and corresponding fitting curve of PVDF@PAN composite SPE with 20wt% PAA.
