# Supplementary material for: Three-Dimensional-Printed Polymer–Polymer Composite Electrolytes for All-Solid-State Li Metal Batteries
Source: Polymers (Basel). 2025 Aug 30;17(17):2369. doi: 10.3390/polym17172369 (PMC12431134; doi:10.3390/polym17172369)
Supplement: Supplementary file 1 [file polymers-17-02369-s001.zip › polymers-3833475-supplementary.pdf]

Supporting information

Three-Dimensional-Printed Polymer–Polymer Composite Electrolytes for All-Solid-State Li Metal Batteries

Hao Wang [1], Xin Xiong [1], Huie Hu [1] and Sijie Liu [2,3,*]

[1] Foundation Department, Naval University of Engineering, Wuhan 430033, China; 1920191001@nue.edu.cn (H.W.); 13971444942@163.com (X.X.); 0907042003@nue.edu.cn (H.H.)
[2] Research Institute of Tsinghua University in Shenzhen, Shenzhen 518000, China
[3] Institute of Nuclear and New Energy Technology, Tsinghua University, Beijing 100084, China
* Correspondence: liusijie@mail.tsinghua.edu.cn

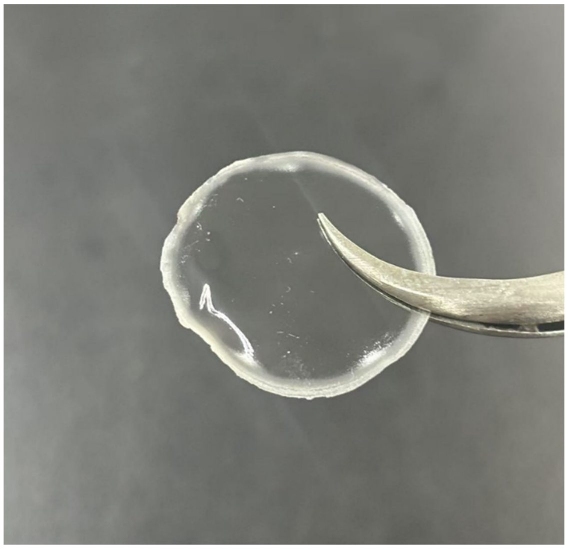

Figure S1. The photo of 3D-printed PVDF membranes.

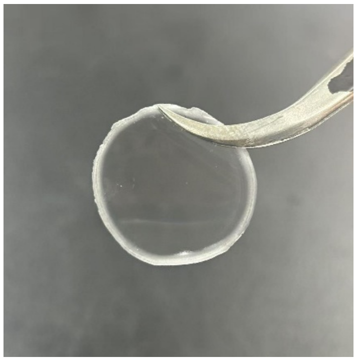

Figure S2. The photo of 3D-printed PVDF@PAN membranes with 1wt% PAA.

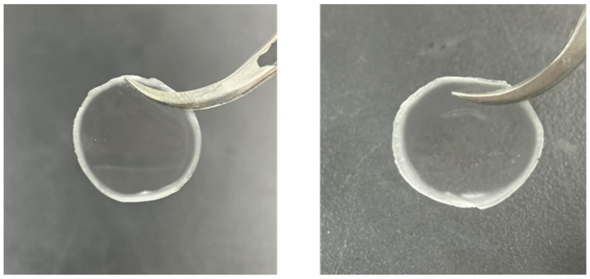

Figure S3. The photo of 3D-printed PVDF@PAN membranes with 3wt% PAA.

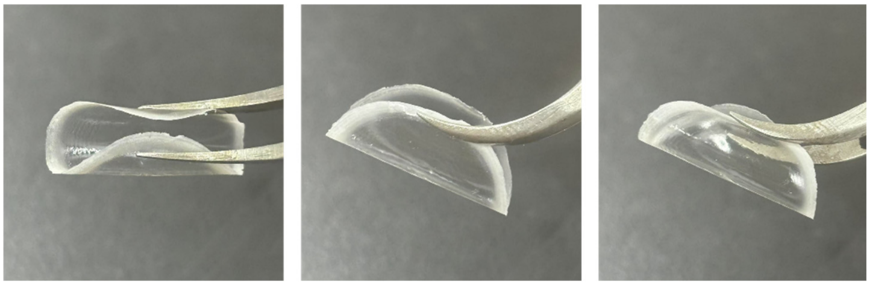

Figure S4. The photo of bendable 3D-printed PVDF@PAN membranes with 3wt% PAA.

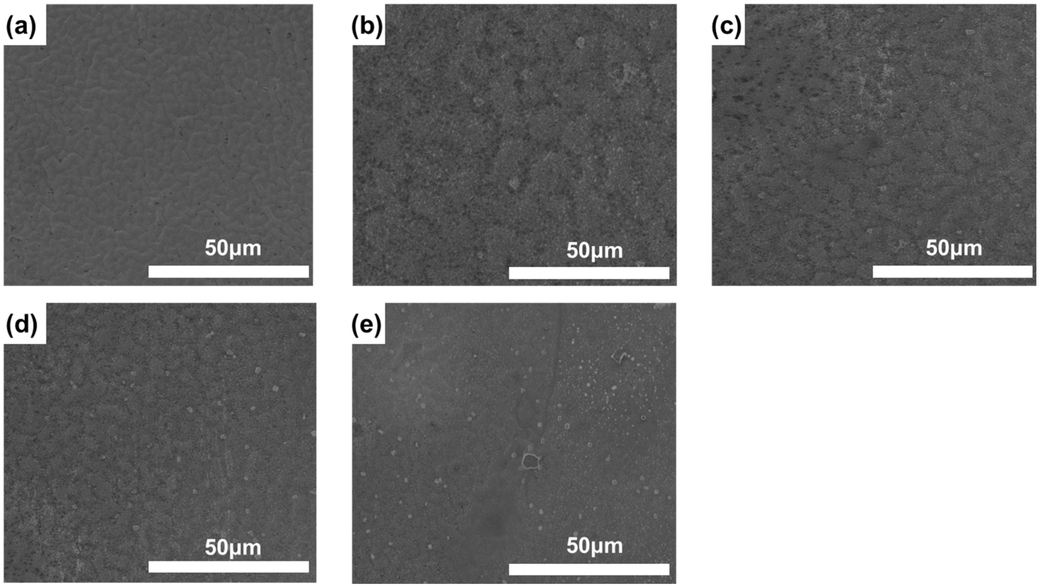

Figure S5. (a)-(e) SEM images of PVDF SPE and PVDF@PAN composite SPEs with different PAA contents (1wt%, 3wt%, 5wt% and 20wt%).

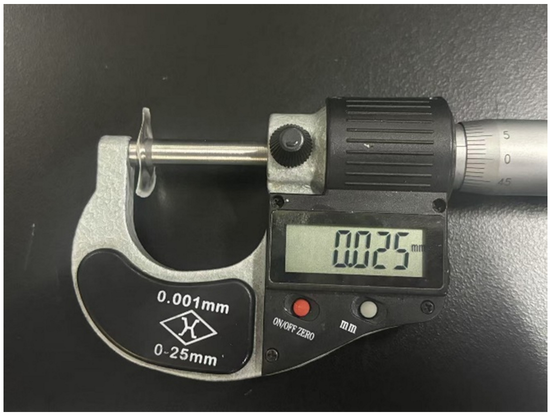

Figure S6. The photo of thickness test for 3D-printed PVDF@PAN membranes with 3wt% PAA.

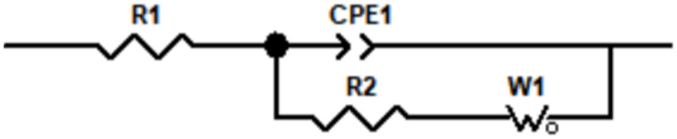

Figure S7. The equivalent circuit used in impedance spectrum fitting.

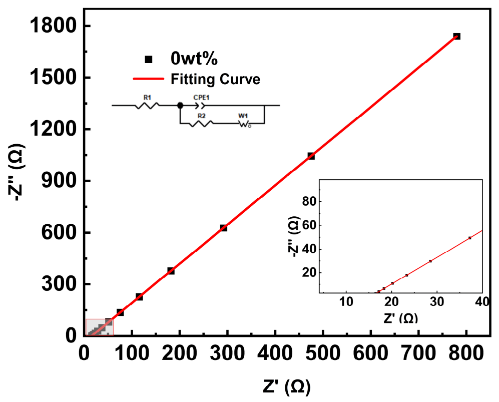

Figure S8. Nyquist spectra and corresponding fitting curve of PVDF SPE (namely 0wt% PAA).

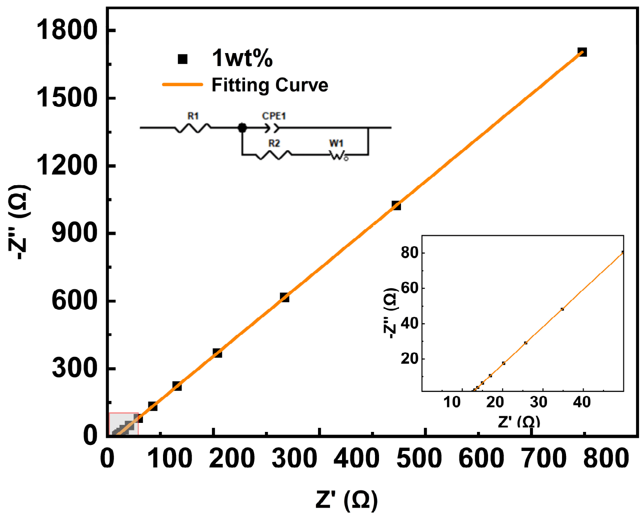

Figure S9. Nyquist spectra and corresponding fitting curve of PVDF@PAN composite SPE with 3wt% PAA.

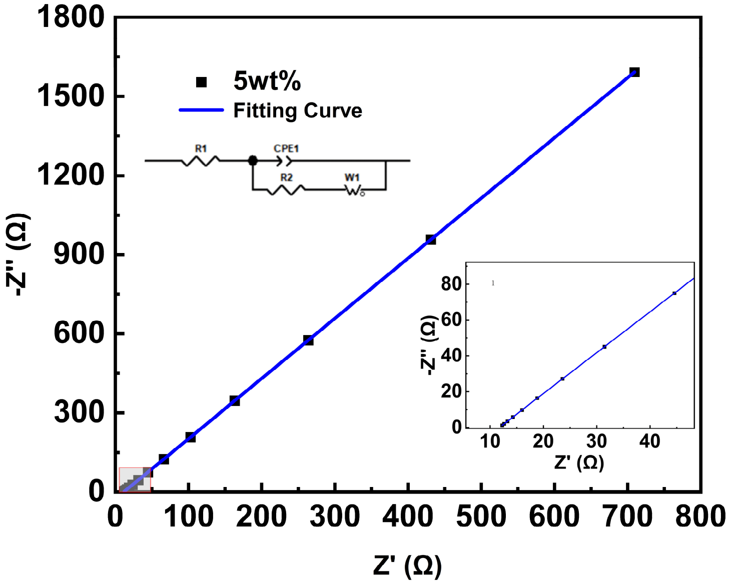

Figure S10. Nyquist spectra and corresponding fitting curve of PVDF@PAN composite SPE with 5wt% PAA.

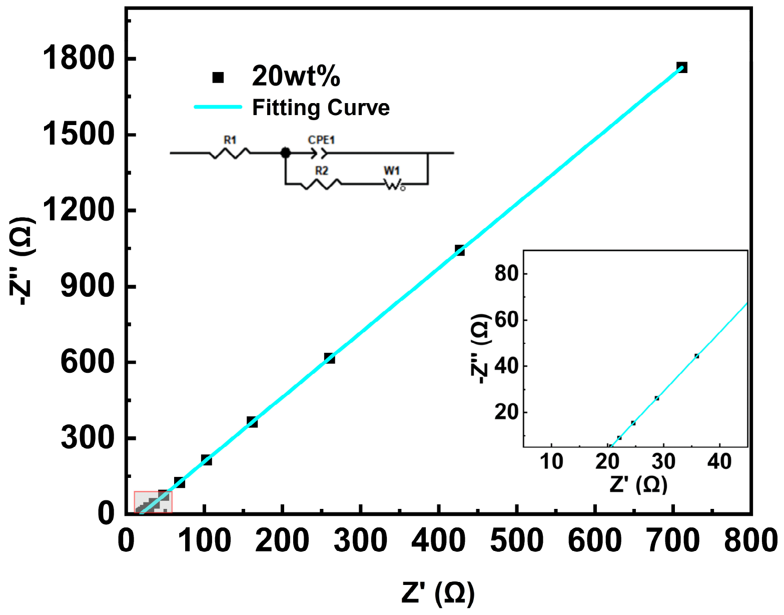

Figure S11. Nyquist spectra and corresponding fitting curve of PVDF@PAN composite SPE with 20wt% PAA.
